# *SLCO1B1 *rs4149056 polymorphism associated with statin-induced myopathy is differently distributed according to ethnicity in the Brazilian general population: Amerindians as a high risk ethnic group

**DOI:** 10.1186/1471-2350-12-136

**Published:** 2011-10-12

**Authors:** Paulo CJL Santos, Renata AG Soares, Raimundo M Nascimento, George LL Machado-Coelho, José G Mill, José E Krieger, Alexandre C Pereira

**Affiliations:** 1 Laboratory of Genetics and Molecular Cardiology, Heart Institute (InCor), University of Sao Paulo Medical School, (Av. Dr. Enéas de Carvalho Aguiar, 44), Sao Paulo, (05403-000), Brazil; 2 Department of Medicine, Ouro Preto Federal University, (R. Diogo de Vasconcelos, 122), Ouro Preto, (35420-000), Brazil; 3 Department of Physiology, Espirito Santo Federal University, (Av. Marechal Campos, 1468), Vitoria, (29040-577), Brazil

**Keywords:** *SLCO1B1*, rs4149056, statins, myopathy, Amerindian, pharmacogenetic

## Abstract

**Background:**

Recent studies reported the association between *SLCO1B1 *polymorphisms and the development of statin-induced myopathy. In the scenario of the Brazilian population, being one of the most heterogeneous in the world, the main aim here was to evaluate *SLCO1B1 *polymorphisms according to ethnic groups as an initial step for future pharmacogenetic studies.

**Methods:**

One hundred and eighty-two Amerindians plus 1,032 subjects from the general urban population were included. Genotypes for the *SLCO1B1 *rs4149056 (c.T521C, p.V174A, exon 5) and *SLCO1B1 *rs4363657 (g.T89595C, intron 11) polymorphisms were detected by polymerase chain reaction followed by high resolution melting analysis with the Rotor Gene 6000^® ^instrument.

**Results:**

The frequencies of the *SLCO1B1 *rs4149056 and rs4363657 C variant allele were higher in Amerindians (28.3% and 26.1%) and were lower in African descent subjects (5.7% and 10.8%) compared with Mulatto (14.9% and 18.2%) and Caucasian descent (14.8% and 15.4%) ethnic groups (p < 0.001 and p < 0.001, respectively). Linkage disequilibrium analysis show that these variant alleles are in different linkage disequilibrium patterns depending on the ethnic origin.

**Conclusion:**

Our findings indicate interethnic differences for the *SLCO1B1 *rs4149056 C risk allele frequency among Brazilians. These data will be useful in the development of effective programs for stratifying individuals regarding adherence, efficacy and choice of statin-type.

## Background

Statins, inhibitors of 3-hydroxy-3-methylglutaryl-coenzyme A (HMG-CoA), are widely prescribed drugs to reduce cardiovascular morbidity and mortality and their use is well tolerated by the majority of patients [1,2]. Nonetheless, statins can lead to myopathy (i.e., muscle pain) with symptoms ranging from mild myalgia without elevated serum creatine kinase to fatal rhabdomyolysis (i.e., muscle breakdown and myoglobin release) [2,3]. In clinical practice, the incidence of mild statin-induced side effects (about 5-10%) appears to be greater than in controlled trials and the mechanisms by which statins lead to myopathy remain unclear. However, studies have suggested that blood statin concentrations, daily dose, female gender, hepatic or renal dysfunction, and concomitant drugs are all predictors of statin-induced myopathy [4-7].

In addition, some recent studies reported the association between *SLCO1B1 *polymorphisms and the development of musculoskeletal side effects [8-10]. The *SLCO1B1 *gene (Gene ID: 10599) encodes the organic anion-transporting polypeptide 1B1 (OATP1B1) that plays a crucial role in the hepatic uptake and clearance of albumin-bound organic compounds [11]. In particular, the *SLCO1B1 *rs4149056 functional polymorphism (c.T521C, p.V174A) in exon 5, leads to higher statin circulating concentration [12,13]. *SLCO1B1 *rs4149056 was associated with an odds ratio for myopathy of 4.5 (95% CI, 2.6 to 7.7) per copy of the C allele and 16.9 (95% CI, 4.7 to 61.1) among CC homozygotes as compared with the TT genotype, in a genome-wide association study plus replication in a trial of simvastatin 40 mg daily involving 20,000 participants [9]. The frequencies of *SLCO1B1 *polymorphisms vary significantly among different world-wide populations, leaving the impact of pharmacogenetic testing for these variants extremely dependent on a particular population genetic architecture.

The Brazilian population is one of the most heterogeneous in the world, being a mixture of different ethnic groups, composed mainly of European (Caucasian descent), African descent and Amerindians [14,15]. Thus, the main aim of the present study was to evaluate the *SLCO1B1 *polymorphisms according to ethnic groups as an initial step for future pharmacogenetic studies and screening programmes.

## Methods

### Study Population

This study included 1,032 subjects of the general urban population selected from the Hearts of Brazil Project (HBP) [16]. The universe of the HBP consisted in the set of inhabitants of Brazilian urban centers with more than 100,000 inhabitants. The HBP sample plan was calculated as 2,500 interviews, distributed in 72 cities from the 5 regions of the country proportionally to the number of inhabitants, per sex and age range, based on data from IBGE (Brazilian Institute of Geography and Statistic). In the selected cities, the "households" constituted the second-stage units, with one interview per household. Subjects were separated in self-identified sub-groups according to ethnicity, as Caucasian descent, African descent, or Mulattos (considered mixed ethnic subjects) [16]. In addition, one hundred and eighty-two Amerindians derived from two different groups (Guarani and Tupinikin; Aracruz Indian Reserve, Espirito Santo State in the Southeast Brazilian coast) were also analyzed. The study protocol was approved by the involved Institutional Ethics Committees and National Ethic Committee for Human Research (CONEP Register Number 4599), and written informed consent was obtained from all participants prior to entering the study.

### Genotyping

Genomic DNA from subjects was extracted from peripheral blood following standard salting-out procedure [17]. Genotypes for the *SLCO1B1 *rs4149056 (c.T521C, p.V174A, exon 5) and *SLCO1B1 *rs4363657 (g.T89595C, intron 11) polymorphisms were detected by polymerase chain reaction (PCR) followed by high resolution melting (HRM) analysis with the Rotor Gene 6000^® ^instrument (Qiagen, Courtaboeuf, France) [18-20]. The QIAgility^® ^(Qiagen, Courtaboeuf, France), an automated instrument, was used according to instructions to optimize the sample preparation step (approximately 30 minutes). One specific disc is able to genotype 96 samples for these polymorphisms.

Amplification of the fragments was performed using the primer sense 5'-TTGTTTAAAGGAATCTGGGTCA-3' and antisense 5'-GAGTCTCCCCTATTCCACGA-3' (77 pairs base) for the rs4149056; and sense 5'-GAGTCCTTCTTTCTCAATTTTTCA-3' and antisense 5'-AAAAGCCCTAGACCAAATGC-3' (107 pairs base) for the rs4363657. A 35-cycle PCR was carried out with the following conditions: denaturation of the template DNA for first cycle of 94°C for 120 s, denaturation of 94°C for 20 s, annealing of 53.4°C (rs4149056) or of 50.5°C (rs4363657) for 20 s, and extension of 72°C for 22 s. PCR was performed using a 10 μL reactive solution (10 mM Tris-HCl, 50 mM KCl, pH 9.0; 2.0 mM MgCl_2_; 200 μM of each dNTP; 0.5 U Taq DNA Polymerase; 200 nM of each primer; 10 ng of genomic DNA template) with addition of fluorescent DNA-intercalating SYTO9^® ^(1.5 μM; Invitrogen, Carlsbad, USA).

**Figure 1:**
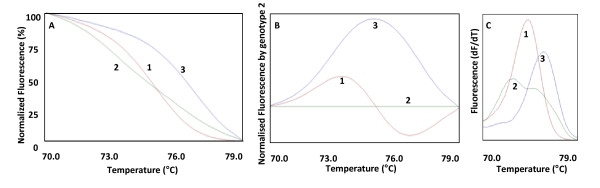
**Graphs of the *SLCO1B1 *rs4149056 (c.T521C, exon 5) nucleotide changes results in different curve patterns using high resolution melting analysis**. A: Graph of normalized fluorescence by temperature. B: Graph of normalized fluorescence (based on genotype 2) by temperature. C: Graph of melting curve analysis (fluorescence differential/temperature differential). 1: wild-type genotype (TT); 2: heterozygous genotype (TC); 3: homozygous genotype (CC) for the *SLCO1B1 *rs4149056 polymorphism. The curve patterns for the *SLCO1B1 *rs4363657 polymorphism (g.T89595C, intron 11) were similar to curve patterns of rs4149056.

In the HRM phase, the Rotor Gene 6000^® ^measured the fluorescence in each 0.1°C temperature increase in the range of 70-79°C (rs4149056) or of 68-77°C (rs4363657). Melting curve were generated by the decrease in fluorescence with the increase in the temperature; and in analysis, nucleotide changes result in three different curve patterns (Figure 1). Samples of the three observed curves were analyzed using bidirectional sequencing as a validation procedure (ABI Terminator Sequencing Kit^® ^and ABI 3500XL Sequencer^® ^- Applied Biosystems, Foster City, CA, USA). The two methods gave identical results in all tests. The wild-type, heterozygous and mutant homozygous genotypes for the rs4149056 and rs4363657 could be easily discernible by HRM analysis. In addition, 4% of the samples were randomly selected and reanalyzed as quality controls and gave identical results.

### Statistical Analysis

Chi-square test was performed for comparative analysis of the *SLCO1B1 *polymorphism frequencies according to ethnicity (African descent, Mulatto, Caucasian descent, and Amerindian). Chi-square test was also performed for the comparative analysis between our data and previous reports. Hardy-Weinberg equilibrium and linkage disequilibrium analyses were conducted with Haploview 4.0. All statistical analyses were carried out using the SPSS software (v. 16.0), with the level of significance set at p < 0.05.

## Results

### General data of the studied population sample

Of the 1,214 subjects (mean age 43.1 ± 14.2), 647 (53.3%) were female and 567 (46.7%) male. Ethnic group distribution was: African descent 8.0% (n = 97), Mulatto 27.3% (n = 332), Caucasian descent 49.7% (n = 603), and Amerindians 15.0% (n = 182).

### Frequencies of the *SLCO1B1 *polymorphisms

The frequencies of the *SLCO1B1 *rs4149056 C variant allele and of the homozygous genotype (CC) were higher in Amerindians (28.3% and 9.9%) and were lower in African descent subjects (5.7% and 0%) compared with Mulatto (14.9% and 2.4%) and Caucasian descent (14.8% and 4.1%) ethnic groups (p < 0.001 and p < 0.001, respectively) (Table 1).

**Table 1:** Allelic and genotypic frequencies for the *SLCO1B1 *polymorphisms according to ethnic groups

Nucleotide Change and Genotype	African descent	Mulatto	Caucasian descent	Amerindian	*p *value
n (100%)	97	332	603	182	
***SLCO1B1 *rs4149056**					
TT	86 (88.7%)	241 (72.6%)	449 (74.5%)	97 (53.3%)	< 0.001
TC	11 (11.3%)	83 (25.0%)	129 (21.4%)	67 (36.8%)	
CC	0	8 (2.4%)	25 (4.1%)	18 (9.9%)	
C allele	5.7%	14.9%	14.8%	28.3%	< 0.001

***SLCO1B1 *rs4363657**					
TT	76 (78.4%)	216 (65.1%)	433 (71.8%)	99 (54.4%)	< 0.001
TC	21 (21.6%)	111 (33.4%)	154 (25.5%)	71 (39.0%)	
CC	0	5 (1.5%)	16 (2.7%)	12 (6.6%)	
C allele	10.8%	18.2%	15.4%	26.1%	< 0.001

*SLCO1B1 *rs4149056: c.T521C, p.V174A (exon 5); *SLCO1B1 *rs4363657: g.T89595C (intron 11).

Mulatto group was composed of mixed ethnicity subjects.

*p *values were calculated using Chi-square test.

The frequencies of the *SLCO1B1 *rs4363657 C variant allele and of the homozygous genotype (CC) were also higher in Amerindians (26.1% and 6.6%) and were lower in African descent subjects (10.8% and 0%) compared with Mulatto (18.2% and 1.5%) and Caucasian descent (15.4% and 2.7%) ethnic groups (p < 0.001 and p < 0.001, respectively) (Table 1).

The genotypic distributions for the *SLCO1B1 *rs4149056 and rs4363657 polymorphisms were in Hardy-Weinberg equilibrium according to ethnic groups.

### Linkage disequilibrium analysis between *SLCO1B1 *rs4149056 and rs4363657 variant alleles according to ethnic groups

**Figure 2:**
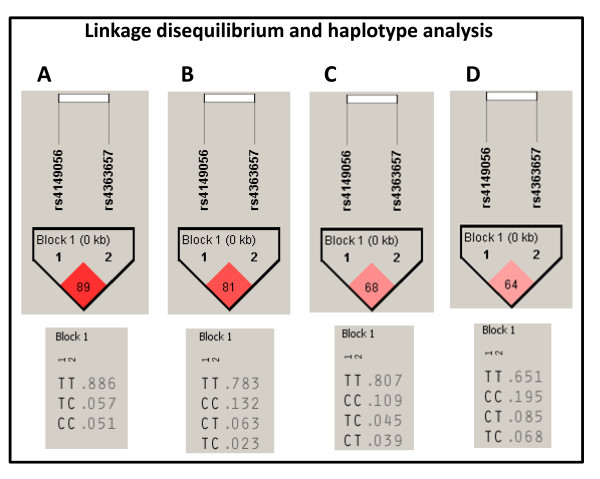
**Linkage disequilibrium and haplotype analysis for the *SLCO1B1 *rs4149056 and rs4363657 polymorphisms in the African descent (A), Mulatto (B), Caucasian descent (C), and Amerindian (D) groups**.

Linkage disequilibrium analysis show that the *SLCO1B1 *rs4149056 and rs4363657 variant alleles are in different linkage disequilibrium patterns depending on ethnic origin (African descent: 89; Mulatto: 81; Caucasian descent: 68; Amerindian: 64) (Figure 2).

## Discussion

The main finding of the present study was to observe significant differences between the allelic and genotypic frequencies of *SLCO1B1 *rs4149056 polymorphism associated with statin-induced myopathy, according to ethnicity in the Brazilian general population. To our knowledge this is the first study indicating that Brazilian Amerindians present higher frequencies of rs4149056 C variant allele (28.3%) and probably higher risk for statin-induced myopathy. These findings could be useful in the strategic planning of the implementation of pharmacogenetic testing according to ethnicity.

The *SLCO1B1 *rs4149056 C variant allele frequency found in our study was higher (p < 0.001) in Amerindian subjects (28.3%) compared with other ethnic groups and with Turkish [21] (12.2%); English [9] (13.0%); European American [22] (14.3%); and German [21] (15.0%) subjects. However, the Caucasian descent (14.8%) and Mulatto (14.9%) ethnic groups of our study presented similar (p > 0.050) rs4149056 C variant allele frequency compared with Caucasian descent subjects from Europe and USA. In addition, our African descent (5.7%) subject group had similar (p > 0.050) frequency compared with African American [22] (2.3%), Uganda African [21] (3.9%), and Tanzania [23] (6.0%) subjects. Interestingly, the frequency observed for the Mulatto subjects indicates that the Amerindian ancestry may be present in this multi-ethnic group, besides the presence of known African ancestry.

For the *SLCO1B1 *rs4363657 polymorphism, C allele frequency found in Caucasian descent in our study (15.4%) was similar (p > 0.050) to that reported in English subjects (13.0%) who participated as controls in a genome-wide association study (GWAS) [9]. The non-coding rs4363657 polymorphism was in nearly complete linkage disequilibrium (LD = 97) with the non-synonymous rs4149056 polymorphism, in the population sample in which the cited GWAS was performed [9]. In our population sample plus Amerindians, it is possible to observe that the *SLCO1B1 *rs4149056 and rs4363657 are in different linkage disequilibrium patterns depending on the ethnic origin. The African descent (LD = 89) and Mulatto (LD = 81) ethnic groups present higher degree of linkage disequilibrium. This highlights two main points: first, even with the different linkage disequilibrium among ethnic groups, the haplotype analysis are not need because only the rs4149056 polymorphism have been described as functional [11,24]. Second, the different frequencies among ethnic groups for the polymorphisms used as markers in the GWAS platforms are limitations/peculiarities of the GWAS methodology [25], which may be acting as interferents in replication studies, mainly in countries with admixed population.

The association between *SLCO1B1 *rs4149056 with myopathy (odds ratio: 4.5 - 95% CI 2.6 to 7.7, per copy of the C allele; and odds ratio: 16.9 - 95% CI 4.7 to 61.1, among CC homozygotes as compared with TT genotypes) was observed in 85 subjects with myopathy taking 80 mg of simvastatin daily as part of a trial involving 12,000 participants; plus replication in a trial of 40 mg of simvastatin daily involving 20,000 participants [9]. The authors of this GWAS plus replication study concluded that common variants in the *SLCO1B1 *gene are strongly associated with an increased risk of statin-induced myopathy and genotyping may help to obtain benefits of statin therapy more safely and effectively [9].

Other studies were performed to replicate this association. Brunham et al observed that rs4149056 genotype was significantly associated (odds ratio 2.3 per C allele) with myopathy in patients receiving simvastatin (mean ~30 mg daily), but not in patients who received atorvastatin [8]. In this way, genotype CC individuals compared with wild-type TT individuals, the area under the plasma concentration time curve for simvastatin acid was increased 221% and for atorvastatin was increased 145% [12,13]. Voora et al confirmed, in 99 subjects with statin-related adverse events, the association previously identified, but also expanded this finding to the most commom statin-induced side effects (e.g., myalgia or muscle ache without significant creatine kinase elevations) [10]. Puccetti et al reported the same association, but in a group of 46 subjects affected by familial hypercholesterolemia treated with atorvastatin and developing myopathy [26].

There are potential limitations in our study. First, the self-referred ethnicity could be creating wrong separation. However, this form of classification is the one most probable to be encountered in real-life situations in the clinical practice. In addition, even self-referred ethnicity was able to clearly differentiate groups of individuals with rather different allele and genotype frequencies. Second, in order to implement a genotyping program in this context, some points could still be evaluated, for example: other genetic markers, primary disease, use of concomitant medications, types of statins, age and gender [11].

## Conclusion

Our findings indicate interethnic differences for the *SLCO1B1 *rs4149056 C risk allele frequency among Brazilian general population plus Amerindians. Recent studies indicated the genotyping for this functional polymorphism as an important clinical tool in future patient-tailored programs. Data of the present study will be useful in the development of effective programs for stratifying individuals regarding adherence, efficacy and choice of statin-type for patients who need statin, specially in high-dose (e.g., familial hypercholesterolemia).

## Competing interests

The authors declare that they have no competing interests.

## Authors' contributions

PCJLS carried out the molecular genetic studies, statistical analysis and drafted the manuscript. RAGS carried out the molecular genetic studies. ACP participated in the design of the study, statistical analysis and manuscript preparation. RMN, GLLMC, JGM, JEK participated in the design of the study and were responsible for individual selection and characterization. All authors contributed critically to the manuscript, whose present version was read and approved by all.

## Pre-publication history

The pre-publication history for this paper can be accessed here:

http://www.biomedcentral.com/1471-2350/12/136/prepub
